# Arthroscopic Stabilization of the Hypermobile Lateral Meniscus Using an All‐Inside Repair Technique

**DOI:** 10.1002/atn2.70338

**Published:** 2026-10-14

**Authors:** Alberto Grassi, Tosca Cerasoli, Vito Gaetano Rinaldi, Giulio Maria Marcheggiani Muccioli, Claudio Rossi, Stefano Zaffagnini

**Affiliations:** ^1^ Department of Biomedical and Neuromotor Sciences DIBINEM Alma Mater Studiorum University of Bologna Bologna Italy; ^2^ 2nd Orthopaedic and Traumatologic Clinic IRCCS Rizzoli Orthopaedic Institute Bologna Italy

## Abstract

The hypermobile lateral meniscus is an uncommon cause of posterolateral knee pain and mechanical symptoms that often escapes standard imaging and is best confirmed by arthroscopy. A clinical stress maneuver combining knee flexion, valgus stress, and external rotation is described in this Technical Note as a simple adjunct to reproduce symptoms related to posterior horn instability and support early suspicion of hypermobile lateral meniscus. This Technical Note also describes a minimally invasive arthroscopic stabilization technique using sequential all‐inside vertical sutures. The procedure begins with fixation of the lateral meniscus to the posterior capsule at the popliteal hiatus, followed by progressive posterior and medial stabilization to restore meniscocapsular integrity while preserving the physiological mobility of the posterior horn. Intraoperative assessment is performed using probing and aspiration tests to avoid overconstraint, and the technique is completed with marrow stimulation in the intercondylar notch to enhance biological healing. This approach restores stability while maintaining native meniscal biomechanics, providing a reproducible and tissue‐preserving strategy for the treatment of hypermobile lateral meniscus.

## Graphical abstract

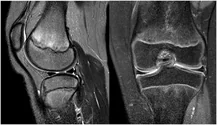

**VIDEO 1:** Arthroscopic all‐inside stabilization of a hypermobile lateral meniscus. The patient is positioned supine with the operative knee placed in the figure‐of‐4 position. Standard anterolateral viewing and anteromedial working portals are established. Diagnostic arthroscopy shows anterior subluxation of the posterior horn of the lateral meniscus during probe examination, consistent with hypermobility. Elevation of the meniscus and inspection of the popliteomeniscal fascicles reveal the insufficiency of the posterolateral meniscal restraints without evidence of a discrete meniscal tear. Visualization through the popliteal hiatus shows an increased distance between the popliteus tendon and the lateral border of the meniscus. Sequential all‐inside vertical sutures are then placed to stabilize the posterior horn. The first stitch is positioned at the popliteal hiatus to provide an immediate reduction of excessive meniscal translation. Additional vertical sutures are sequentially placed more medially according to the residual instability identified during arthroscopic assessment. During tensioning, a probe is interposed between the suture and the meniscus to protect the tissue, avoid overconstraint, and preserve a small degree of physiological posterior horn mobility. Stability is reassessed after each fixation point, and sutures are cut once optimal stabilization has been achieved. Final probing confirms the restoration of stability and reduction of the gap between the popliteus tendon and the lateral meniscus. The procedure is completed with marrow stimulation of the intercondylar notch using 3 microperforations to enhance the biological healing environment. Video content can be viewed at https://doi.org/10.1002/atn2.70338.

The hypermobile lateral meniscus (HLM) is a rare yet likely underdiagnosed cause of knee pain.^1^ It is known that the lateral meniscus is physiologically more mobile than its medial counterpart because of its thinner, laxer posterior capsule and the interruption of capsular attachments at the popliteal hiatus, features that predispose to excessive translation, especially in deep flexion.^2^, ^3^, ^4^ Pathological hypermobility arises when the stabilizing structures of the posterolateral meniscus fail. Several conditions may underlie this instability. These include idiopathic HLM, in which insufficiency of the popliteomeniscal fascicles (PMFs), meniscotibial ligaments, and meniscocapsular restraints allows abnormal posterior horn translation even in the absence of major trauma, particularly in young patients.^5^ Other causes are traumatic tears of the PMFs and congenital absence of meniscotibial and capsular attachments, a condition known as the Wrisberg‐type III meniscus, in which the meniscofemoral ligaments of Humphrey and Wrisberg represent the only posterior stabilizers.^6^, ^7^ Regardless of cause, the literature consistently reports similar presenting symptoms, such as locking sensation, knee pain, snapping, giving way, and clicking, frequently associated with a normal or nondiagnostic magnetic resonance imaging (MRI).^7^, ^8^, ^9^ Definitive diagnosis is therefore arthroscopic: in the figure‐of‐4 position, aspiration or probing elicits anterior translation of the posterior horn exceeding half the width of the lateral tibial plateau, often associated with “crescent‐moon” exposure of the lateral tibial cartilage at the popliteal hiatus.^2^, ^3^ Arthroscopy also allows direct visualization of tears or insufficiencies of the PMFs.^10^ Current evidence supports meniscal preservation, using all‐inside or inside‐out repair techniques. Although favorable clinical outcomes have been reported, there is still no consensus on the optimal number and configuration of sutures, particularly in pediatric athletes.^8^

## SURGICAL TECHNIQUE

### Clinical Diagnosis

Diagnosis of lateral meniscus hypermobility can be challenging because routine clinical examination and MRI are frequently unremarkable. Preoperative MRI may show a morphologically normal lateral meniscus, without discoid configuration, meniscal tear, or obvious signal alteration (Figure 1). Therefore, the absence of clear MRI abnormalities does not exclude HLM.

**FIGURE 1:**
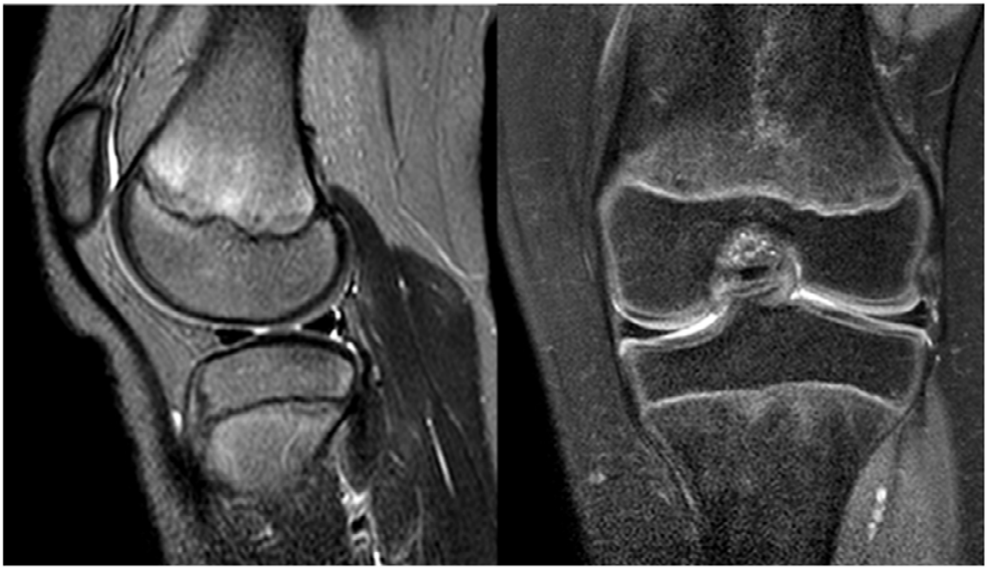
Preoperative MRI revealing a morphologically normal lateral meniscus, without discoid morphology or detectable signal alterations. Fluid collection behind the posterior horn of the lateral meniscus could guide toward the suspect of hypermobile meniscus. (MRI, magnetic resonance imaging.)

Delayed recognition may result in persistent symptoms, progressive instability, and secondary chondral damage, highlighting the importance of early clinical suspicion.^11^, ^12^ In addition to the classic reproduction of symptoms in the figure‐of‐4 position, a provocative stress maneuver has recently been proposed as a useful adjunct. This test consists of knee flexion combined with valgus stress and external rotation of the foot, which may elicit apprehension or discomfort in patients with HLM because of posterior horn subluxation. In contrast, varus stress does not reproduce symptoms. Although not yet universally validated, this maneuver may provide additional clinical clues (Figure 2).

**FIGURE 2:**
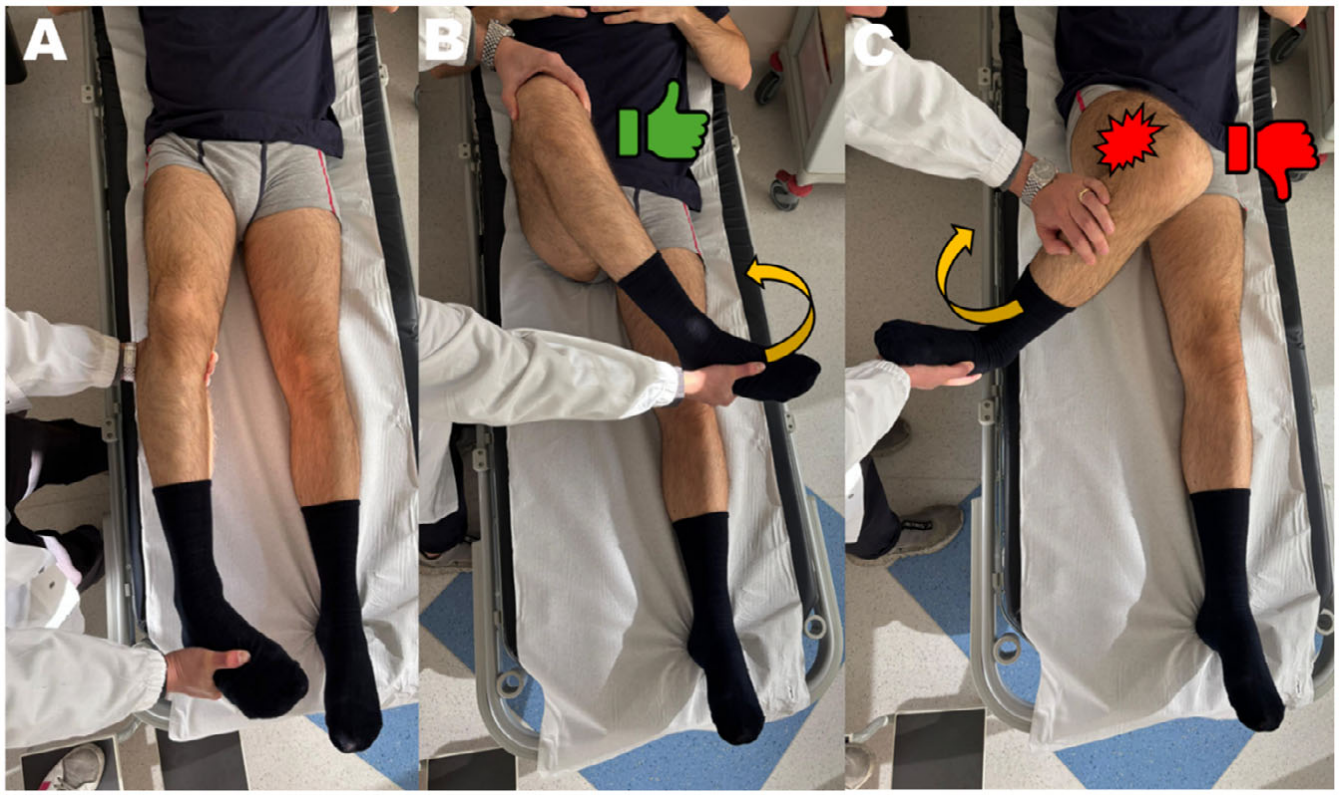
Clinical stress test for HLM. (A) Starting position: patient supine with knee flexion and foot internally rotated. (B) Negative varus stress test, where hip flexion, knee flexion, and foot internally rotated do not provoke apprehension. (C) Positive valgus stress test, where hip flexion, knee flexion, and external rotation of the foot induce apprehension. (HLM, hypermobile lateral meniscus.)

### Operative Technique

After tourniquet application and sterile draping, standard anterolateral and anteromedial portals are established, and a 30° arthroscope is introduced. The procedure can be performed using commercially available all‐inside meniscal repair devices according to surgeon preference and local availability. In the present technique, vertical meniscocapsular sutures are placed using an all‐inside meniscal repair system, such as FAST‐FIX 360 or FAST‐FIX FLEX (Smith & Nephew, Andover, MA). A 30° arthroscope, standard anterolateral and anteromedial portals, arthroscopic probe, shaver, and microfracture awl are also required. After checking the medial compartment, the lateral compartment is assessed through the figure‐of‐4 position to access the popliteal hiatus corridor. The diagnosis of hypermobility is confirmed when probe traction produces anterior translation of the posterior horn greater than 50% of the lateral tibial plateau (Figure 3, Video 1); insufficiency of posterolateral restraints is evidenced by elevating the meniscus and inspecting the PMFs and by visualizing a widened interval between the popliteus tendon and the lateral meniscal border. The aspiration test and “crescent‐moon” sign are documented at baseline.

**FIGURE 3:**
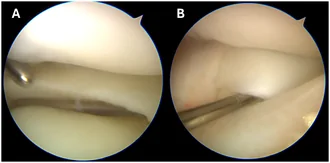
Arthroscopic evaluation of lateral meniscus hypermobility. (A) Lateral meniscus in normal resting position. (B) Anterior probing shows excessive translation beyond the LFC. (LFC, lateral femoral condyle.)

Stabilization proceeds with 3 sequential all‐inside vertical stitches, each tensioned under probe guidance to recover stability while retaining a slight physiologic glide and avoiding damaging the meniscus (as detailed in Video 1). The first stitch links the posterolateral capsule at the popliteal hiatus to the posterior horn of the lateral meniscus just medial to the popliteus without tethering the tendon; this immediately reduces gross anterior translation (Figure 4). The second stitch is placed more posteriorly, beyond the popliteus trajectory, to reinforce the posterior capsular restraint and limit the residual hypertranslation of the horn (Figure 5). The third stitch secures the inferior fibers of the posterior horn to the posterior capsule to address any remaining laxity along the meniscocapsular interface while preserving the physiological insertion of the lateral posterior horn (Figure 6).

**FIGURE 4:**
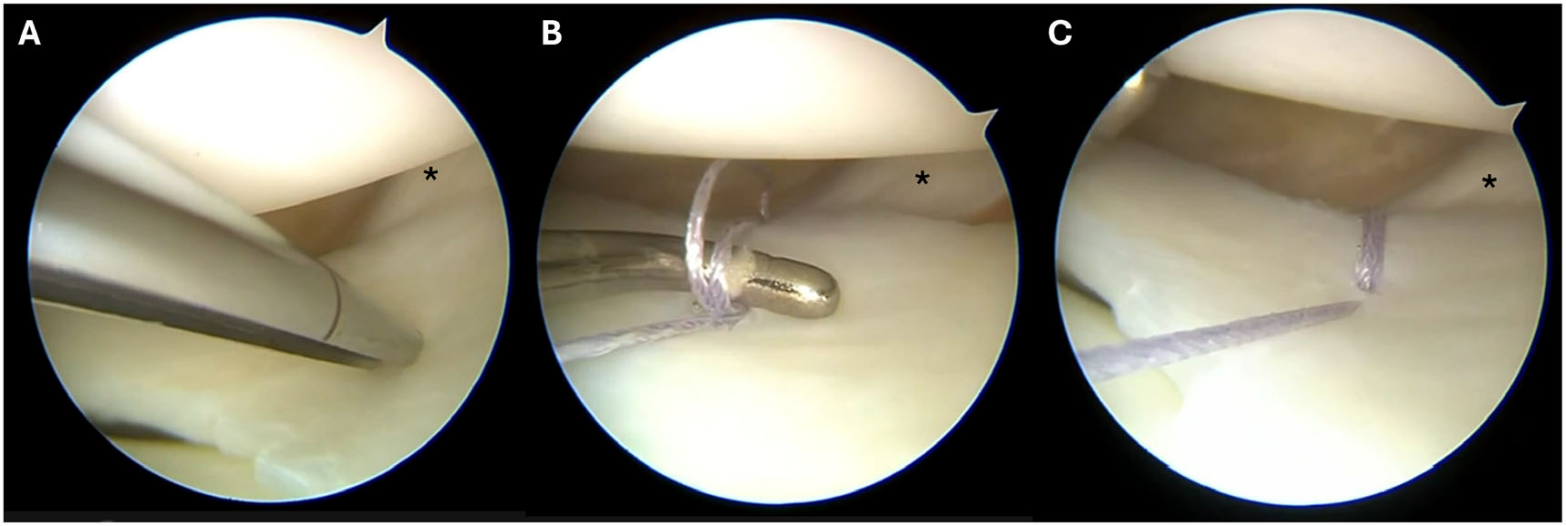
Placement and tensioning of the first stabilization stitch. (A) The first stitch is placed through the posterolateral capsule at the popliteal hiatus. (B) The second fixation point is established at the posterior horn of the lateral meniscus just medial to the popliteus tendon (asterisk). The stitch is progressively tensioned under probe control while preserving free excursion of the popliteus tendon. (C) Arthroscopic appearance after final tensioning of the first stitch.

**FIGURE 5:**
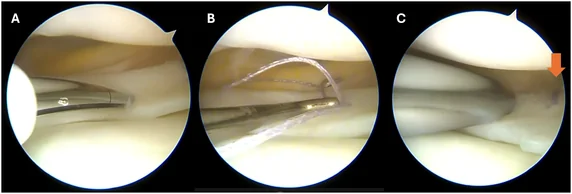
Placement of the second stabilization stitch. (A) The second stitch is positioned superior and more medial than the first fixation point to further stabilize the posterior horn of the lateral meniscus. (B) Vertical orientation of the second all‐inside suture linking the posterior horn to the adjacent capsular tissue. (C) Arthroscopic view during suture trimming. The arrow identifies the first stitch, highlighting the sequential vertical fixation pattern used to progressively reduce posterior horn hypermobility.

**FIGURE 6:**
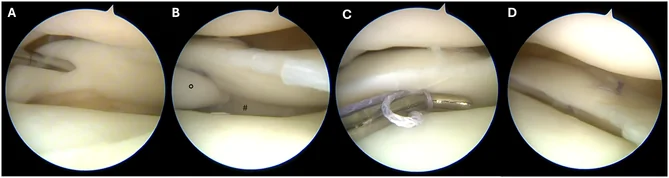
Placement and tensioning of the third stabilization stitch. (A) A third all‐inside device is inserted, and an additional vertical suture is placed at the posterior aspect of the lateral meniscus. (B) Arthroscopic view of the third fixation point. The suture stitch is identified by a circle (°), whereas the posterior capsule is marked with a hash symbol (#), illustrating the meniscocapsular fixation. (C) Probe‐assisted tensioning of the suture. The probe is interposed between the suture and the meniscal tissue to protect the meniscus during tensioning and to avoid excessive tightening that may result in overconstraint. (D) Final appearance of the third stitch after appropriate tensioning and before suture trimming, showing stable fixation while preserving physiological meniscal mobility.

After each pass, the probe is interposed between the suture limbs to protect the meniscal tissue during incremental tensioning. Aspiration and traction tests are repeated to avoid overconstraint; the goal is to restore stability yet maintain a small, palpable glide of the horn against the capsule as similar to physiological as possible (Figure 7). The aspect of the 3 stitches is depicted in Figure 8. When the popliteus‐meniscus interval has visibly narrowed (Figure 9) and instability tests become negative, marrow stimulation through microfractures is performed in the intercondylar notch (Figure 10).

**FIGURE 7:**
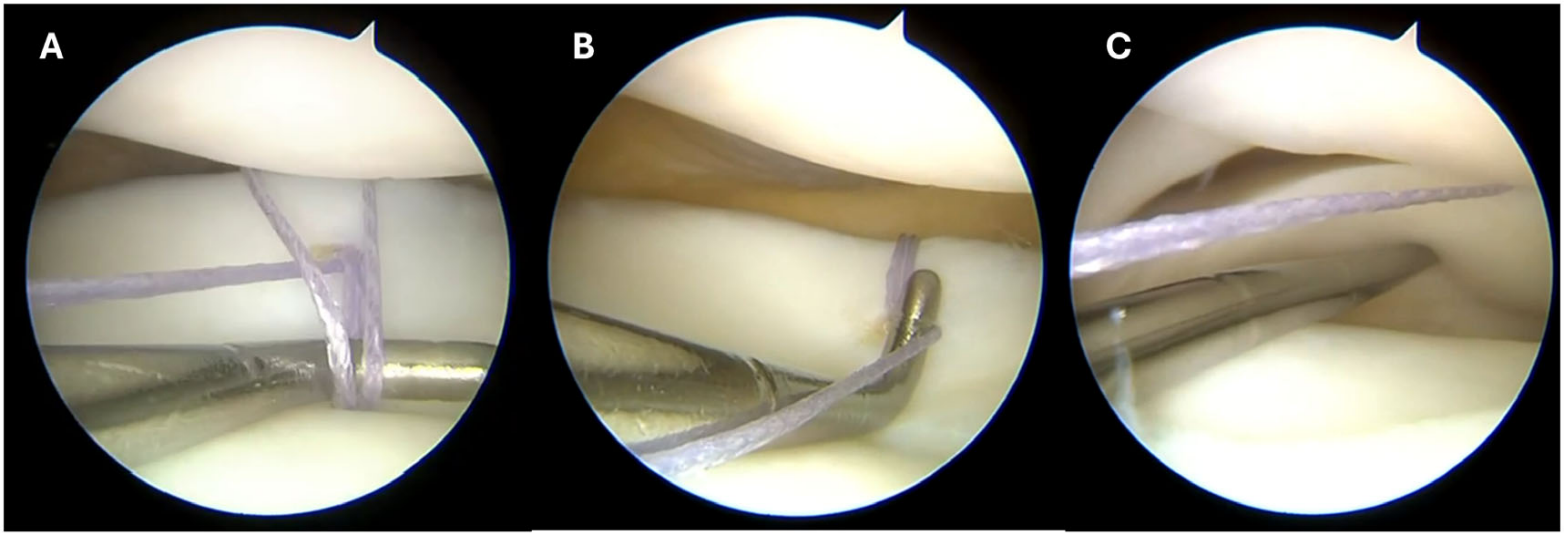
Tensioning of sequential stabilization sutures. (A) The probe is positioned between the suture and the meniscal tissue during tensioning. Because traction is applied to the suture, the force is transmitted against the probe rather than directly to the meniscus, protecting the tissue from excessive compression or cut‐through. (B) Gentle pulling of the suture against the probe allows fine adjustment of the fixation, helping avoid overconstraint of the posterior horn of the lateral meniscus. (C) Final probe assessment before suture trimming. The posterior horn should remain stable while preserving a small degree of physiological excursion (some millimeters), confirming that the meniscus is stabilized but not overconstrained.

**FIGURE 8:**
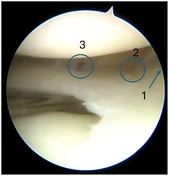
Final configuration of the 3 stabilization sutures. The first stitch is indicated by the arrow, whereas the second and third stitches are circled. The image shows the spacing between fixation points. Suture position and intersuture distance are not standardized and are determined intraoperatively according to the pattern and extent of posterior horn hypermobility.

**FIGURE 9:**
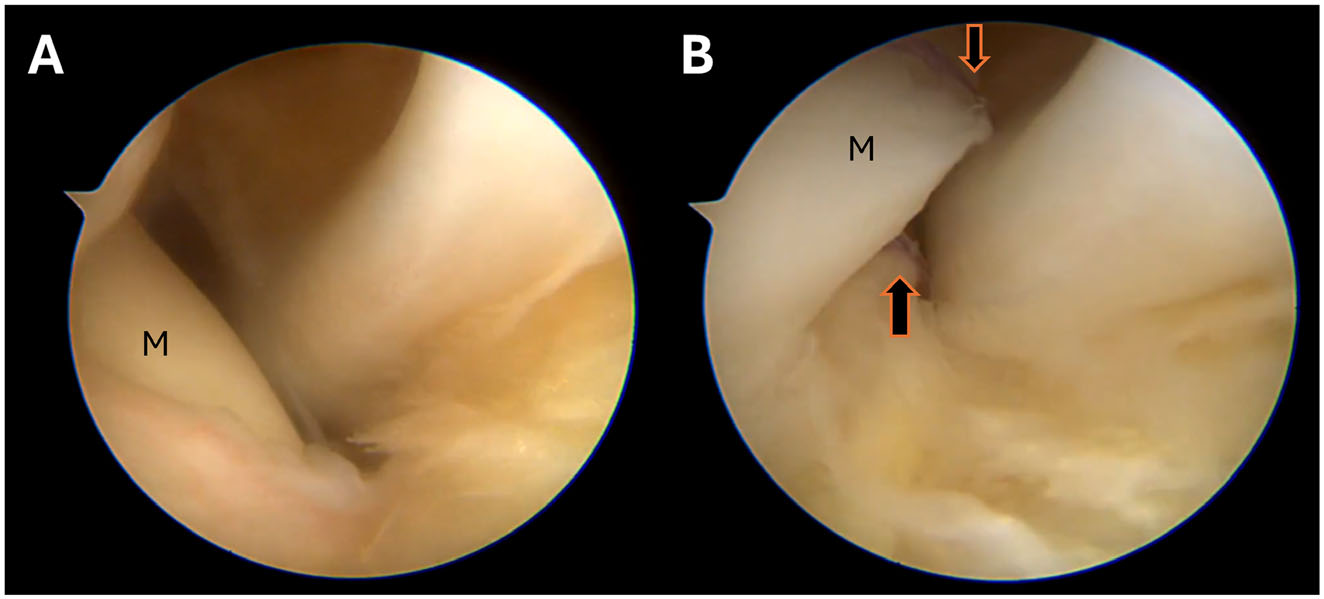
Arthroscopic evaluation of popliteal hiatus. (A) Widened popliteal hiatus before repair and (B) popliteal hiatus after repair, with restored meniscocapsular attachment. The posterior meniscal wall is identified by M. Arrows indicate the stabilization sutures placed during the repair.

**FIGURE 10:**
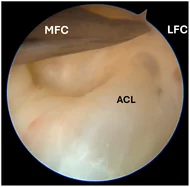
Final marrow stimulation procedure. After restoration of meniscal stability, 3 microperforations are performed within the intercondylar notch to enhance the biological healing environment. (ACL, anterior cruciate ligament; LFC, lateral femoral condyle; MFC, medial femoral condyle.)

A final sweep confirms free popliteus gliding without impingement. Pearls and pitfalls of the technique, as well as indications and contraindications, are summarized in Tables 1 and 2.

**TABLE 1:** Pearls and Pitfalls of All‐Inside Stabilization for the Treatment of HLM: The Table Summarizes Key Technical Pearls and Pitfalls to Optimize Stabilization While Preserving Physiological Meniscal Mobility

**Pearls**	**Pitfalls**
Perform diagnostic assessment in the figure‐of‐4 position to clearly expose the popliteal hiatus and posterior horn mobility	Inadequate evaluation of posterior horn mobility before fixation
Confirm instability dynamically using probing and aspiration before starting fixation	Failure to recognize popliteomeniscal fascicle insufficiency
Begin stabilization at the popliteal hiatus, where excessive translation is usually most evident	Misplacement of sutures too close to the popliteus tendon, risking tethering or impingement
Use sequential vertical sutures to progressively reduce instability rather than attempting immediate rigid fixation	Overtightening sutures leading to loss of physiological mobility or iatrogenic stiffness
Tension sutures under direct probe control to preserve a slight physiological glide of the posterior horn	Incomplete stabilization because of an insufficient number or poor positioning of sutures
Maintain visualization of the popliteus tendon throughout the procedure	Assuming all hypermobile menisci require identical fixation patterns
Reassess stability after each stitch using traction and aspiration tests	Skipping dynamic reassessment after each fixation step
Use the figure‐of‐4 position during fixation to optimize visualization and working angle	Excessive reliance on MRI findings despite normal or inconclusive imaging
Perform marrow stimulation only after final stability has been achieved	Overuse of biologic adjuncts without adequately addressing mechanical instability
Aim for stability without eliminating normal lateral meniscal mobility	Underestimating the learning curve associated with this technique

HLM, hypermobile lateral meniscus; MRI, magnetic resonance imaging.

**TABLE 2:** Indications and Contraindications for All‐Inside Stabilization of the HLM

**Indications**	**Contraindications**
Symptomatic mechanical locking or giving way	Advanced lateral compartment osteoarthritis
Positive figure‐of‐4 or stress maneuver	Irreparable meniscal deficiency
Arthroscopically confirmed hypermobility	Severe capsular deficiency requiring reconstruction
Failure of conservative treatment	Inability to comply with postoperative protection
Pediatric or young active patients	Poor tissue quality preventing secure fixation
Wrisberg‐type or PMF‐related instability	Active infection or inflammatory arthropathy

HLM, hypermobile lateral meniscus; PMF, popliteomeniscal fascicle.

### Postoperative Management

Postoperative care begins with a knee brace in full extension day and night. Exercises for isometric strengthening are immediately allowed to maintain quadriceps tone. Three days after surgery, exercises to maintain the range of motion with a continuous passive motion device are also allowed, increasing flexion by approximately 5° per session until 90° is reached. Crutch‐assisted ambulation is maintained without weight‐bearing until suture removal at about 15 days; then, weight‐bearing is progressed to full, and the brace is discontinued around 30 days with initiation of aquatic physiotherapy. Pivoting and deep flexion are allowed 4 months postoperatively. A graduate return to running precedes sport‐specific drills; adolescent athletes typically resume field activities when asymptomatic and symmetric on functional testing, commonly near 5 months, with timelines individualized to intraoperative tissue quality and end‐of‐procedure stability. This progression is consistent with meniscal‐sparing stabilization principles described for hypermobility and with rehabilitation protocols that protect the meniscocapsular repair while restoring motion.^6^, ^7^

## DISCUSSION

The primary goal in the treatment of HLM is to restore functional stability while preserving the intrinsic mobility of the lateral compartment. Excessive constraint may alter the native knee kinematics and increase contact pressures, whereas insufficient stabilization may lead to persistent mechanical symptoms and recurrent subluxation.^11^, ^12^

For this reason, early recognition and timely treatment are particularly important, especially in young and active patients, to prevent progressive meniscal dysfunction and secondary cartilage damage.^11^ Diagnosis of HLM remains challenging because routine clinical examination and MRI are frequently nondiagnostic.^7^, ^8^, ^10^, ^13^ In this context, we describe a simple stress maneuver combining knee flexion, valgus stress, and external rotation, which may help reproduce symptoms related to posterior horn instability and support early clinical suspicion when imaging findings are inconclusive. Although this maneuver has not yet been formally validated, it represents a practical adjunct to the clinical examination and may facilitate the earlier identification of patients who warrant arthroscopic evaluation.

Several anatomical substrates may underlie HLM; current evidence suggests that the underlying mechanism does not substantially modify the surgical strategy. Reported treatment options range from radiofrequency‐based capsular shrinkage aimed at collagen remodeling^14^ to posterior meniscal root reinsertion using transosseous knotless fixation^15^ and more complex reconstructions of the posterolateral meniscocapsular restraints.^13^ A recent systematic review including more than 200 patients showed that most authors prefer all‐inside or inside‐out repair strategies, reporting high rates of symptom resolution and low recurrence regardless of the specific construct used.^8^ These findings support a minimally invasive approach focused on restoring stability while respecting the native biomechanics of the lateral meniscus.

The technique presented in this study aligns with these principles.^6^, ^7^ Sequential vertical sutures allow progressive control of instability while minimizing the risk of overtightening. Similar concepts have been described in previous reports, in which stabilization is directed toward the posterior capsule, the popliteomeniscal region, or the meniscocapsular junction. The figure‐of‐4 position facilitates the exposure of the lateral compartment and enables dynamic intraoperative assessment of residual mobility. All‐inside suture devices are widely adopted because they provide reliable fixation with limited surgical morbidity. In the present technique, suture tension is carefully adjusted under probe guidance to preserve a slight physiological glide of the posterior horn and to avoid iatrogenic overconstraint or meniscal tissue damage.^8^ Intercondylar notch microfracture is used as a biological adjunct based on broader experience in meniscal repair, although specific evidence supporting its role in HLM remains limited.^16^

This technique offers several advantages. It is fully arthroscopic, avoids posterior open dissection, and relies on widely available all‐inside meniscal repair devices. The sequential placement of vertical sutures allows progressive correction of instability, whereas repeated probing and aspiration testing help avoid overconstraint. Furthermore, the procedure is tissue‐preserving and aims to restore meniscocapsular stability without sacrificing the native meniscal tissue or eliminating the physiological mobility of the lateral meniscus. These characteristics may be particularly advantageous in young and active patients, in whom preservation of native knee biomechanics is of paramount importance.

Nevertheless, some limitations should be acknowledged. Diagnosis remains largely arthroscopic and operator‐dependent, especially in patients with normal or inconclusive MRI findings. In addition, the optimal number, position, and tensioning of sutures have not yet been standardized. Excessive tensioning may overconstrain the posterior horn and alter its physiological motion, whereas insufficient fixation may result in persistent instability. Careful visualization of the popliteus tendon is therefore essential to avoid tethering or iatrogenic impingement during repair. The main advantages and limitations of this technique are summarized in Table 3.

**TABLE 3:** Advantages and Limitations of Arthroscopic Stabilization of the Hypermobile Lateral Meniscus Using an All‐Inside Repair Technique

**Advantages**	**Limitations**
Fully arthroscopic and minimally invasive	Diagnosis remains largely arthroscopic and operator‐dependent
Avoids posterior open dissection and associated surgical morbidity	The procedure is highly surgeon‐dependent, requiring experience in recognizing pathological meniscal hypermobility and balancing restoration of stability with preservation of physiological mobility
Uses widely available all‐inside meniscal repair devices	The optimal number, position, and tension of sutures have not been standardized
Sequential vertical sutures allow progressive stabilization of the posterior horn	Excessive tensioning may overconstrain the posterior horn and alter physiological meniscal mobility
Dynamic probing and aspiration testing guide intraoperative tensioning	Insufficient fixation may result in persistent instability or recurrent symptoms
Preserves native meniscal tissue and physiological lateral meniscal mobility	Careful visualization is required to avoid popliteus tendon tethering or impingement
Particularly suitable for young and active patients in whom meniscal preservation is desirable	Current evidence is limited to technical reports, case reports, and small case series

Finally, the available evidence remains limited, consisting predominantly of case reports, technical notes, and small case series. Further prospective studies are required to establish reproducible diagnostic criteria, define objective intraoperative tensioning parameters, and evaluate mid‐ and long‐term clinical outcomes after surgical stabilization of HLM.

## DECLARATION OF GENERATIVE AI AND AI‐ASSISTED TECHNOLOGIES IN THE WRITING PROCESS

During the preparation of this work, the authors used an artificial intelligence tool (ChatGPT, OpenAI) to assist with English language editing. After using this tool/service, the authors reviewed and edited the content as needed and take full responsibility for the content of the publication.

## DISCLOSURES

The authors (A.G., T.C., V.G.R., G.M.M.M., C.R., S.Z.) declare that they have no known competing financial interests or personal relationships that could have appeared to influence the work reported in this article.

## FUNDING

Open access publishing facilitated by Universita di Bologna, as part of the Wiley ‐ CRUI‐CARE agreement.
